# Medroxyprogesterone acetate levels among Kenyan women using depot medroxyprogesterone acetate in the FEM-PrEP trial^☆^^☆☆^

**DOI:** 10.1016/j.contraception.2016.03.003

**Published:** 2016-07

**Authors:** Kavita Nanda, Rebecca Callahan, Douglas Taylor, Meng Wang, Kawango Agot, David Jenkins, Lut Van Damme, Laneta Dorflinger, for the FEM-PrEP Study Group

**Affiliations:** a FHI 360, Durham, NC 27701, USA; b Impact-RDO, Kisumu, Kenya; c Bill & Melinda Gates Foundation, Seattle, WA 98109, USA

**Keywords:** Depot medroxyprogesterone acetate, Preexposure prophylaxis, HIV, Kenya

## Abstract

**Objective:**

To describe medroxyprogesterone acetate (MPA) levels among Kenyan depot medroxyprogesterone acetate (DMPA) users in the FEM-PrEP HIV prevention trial, and to compare MPA levels between ARV for HIV prevention (treatment) and placebo groups.

**Study Design:**

We measured MPA in previously collected plasma samples from 63 Kenyan trial participants who used DMPA for one or two complete intervals. We separately assessed MPA levels among the nine DMPA users who became pregnant at this site.

**Results:**

Mean MPA levels at the end of each 12 week injection interval were 0.37 ng/ml (95% CI: 0.25, 1.99) and 0.28 ng/ml (95% CI: 0.19, 1.22) among participants assigned TDF/FTC and 0.49 (95% CI: 0.40, 1.27) and 0.39 (95% CI: 0.31, 1.17) among those assigned placebo. The difference between groups was not statistically significant overall, or in an analysis which adjusted for the observed low adherence to TDF/FTC. Unanticipated findings of this analysis were low 12-week MPA levels among DMPA users in both study arms. Of 61 women who contributed data for the first DMPA injection interval, 26.2% had MPA levels < 0.1 ng/ml and 9.8% had levels below the detection level (0.02 ng/ml) at 12 weeks post-injection. Levels were similar at the end of the second injection interval. Five of nine women who became pregnant had levels below 0.15 ng/mL at the time of their last negative pregnancy test.

**Conclusions:**

Use of TDF/FTC did not appear to affect serum MPA levels, however we found lower than expected MPA concentrations at the end of the dosing interval among DMPA users in the FEM-PrEP trial, the cause of which are unknown.

**Implications:**

This study presents some of the few available data on MPA levels among DMPA users in Africa. The low levels among users described here, together with a number of pregnancies among DMPA users, are potentially concerning and require further investigation.

## 1 Introduction

Around the world an estimated 48 million women rely on injectable contraceptives for pregnancy prevention [1], [2]. This figure has increased substantially over the last two decades, especially in lesser developed regions where injectable methods are increasingly available and popular [3], [4]. In Sub-Saharan Africa, nearly half of all modern contraceptive users are using injectable contraceptives [1]. As in the rest of the world, most injectable users in Africa use the three-month progestin-only injectable depot-medroxyprogesterone acetate (DMPA) [4].

Despite the increasing popularity of DMPA, few studies report on pregnancy rates or medroxyprogesterone acetate (MPA) levels among diverse populations in low resource settings. Recently, we noted several unexpected pregnancies among DMPA users in the Bondo, Kenya site of the FEM-PrEP Trial for HIV Prevention among African Women. These pregnancies, along with higher overall pregnancy rates among women randomized to tenofovir/emtricitabine (TDF/FTC) [5], raised concerns that use of the study drug could be interfering with the effectiveness of hormonal contraceptives. Even though TDF/FTC is not known to be a hepatic enzyme inducer, and would not be expected to affect contraceptive efficacy, we were concerned about potential interactions other than hepatic enzyme induction. In response to this concern, we evaluated MPA levels in a sub-sample of DMPA users, and compared MPA levels by study arm. We also evaluated MPA levels in women who conceived while using DMPA.

## 2 Materials and methods

This is a secondary analysis of data from FEM-PrEP, a randomized placebo-controlled trial of daily oral TDF/FTC to prevent HIV acquisition, that enrolled 2120 HIV-negative women aged 18–35 at four sites in South Africa, Kenya and Tanzania. Participants were asked to use study drug (TDF/FTC or placebo) daily for 52 weeks and attend study visits every four weeks. Use of an effective, non-barrier method of contraception, including sterilization, IUD, progestin implants, injectables (DMPA or NET-En), or combined oral contraceptives (COCs) was required at the time of enrollment and encouraged, but not required, at follow-up. The FHI 360 Protection of Human Subjects Committee (PHSC) and institutional review boards at the study sites approved the study protocol, and participants consented to additional investigation of collected blood samples. The trial was stopped early because of lack of effect of TDF/FTC on HIV acquisition, subsequently found to be due to poor adherence. Effectiveness and safety data from FEM-PrEP and other study details have been published previously [5].

**Fig. 1:**
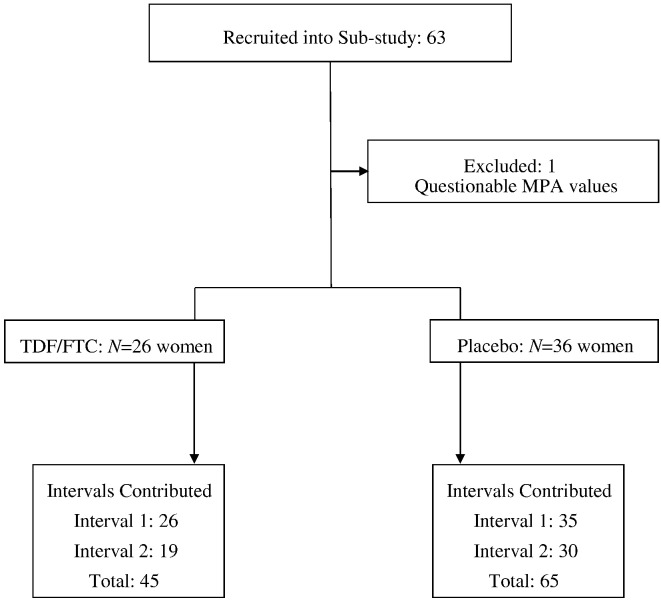
Study population and intervals contributed to analyses.

For this secondary analysis, we evaluated MPA levels in women from the Bondo, Kenya site who: 1) had a DMPA injection at enrollment and 2) completed their week 4, 8 and 12 study visits on schedule. A total of 63 women met these criteria: 11 new DMPA users and 52 continuing users. Of these 63, 49 further completed a second interval of DMPA use and made their week 16, 20 and 24 study visits (Fig. 1). Because of incomplete DMPA injection data from other FEM-PrEP countries, we were only able to evaluate samples from the Kenya site, where injections were given on site by study nurses, We used theses specimens to compare MPA levels between women randomized to TDF/FTC and those randomized to placebo. Sample size was based on the totality of women eligible for the analysis, and not formal power considerations. Nonetheless, our observed sample size (36 and 26, respectively, in the placebo and TDF/FTC groups) provided approximately 76% power to detect a 50% reduction in trough (week 12) MPA concentrations (two-sided alpha = 0.05) among TDF/FTC users, assuming a coefficient of variation of 0.9 (the average observed value), with still greater power for repeated measures (interval) analysis. We separately assessed MPA levels for nine of 12 DMPA users at the Bondo site who tested positive for pregnancy during the study.

Sites recorded demographic and basic physical examination information including height and weight on standardized case report forms at enrollment. At enrollment and each follow-up visit, participants provided blood samples, had urine tested for pregnancy, and reported current contraceptive and concomitant medication use during the previous 4-week interval. In the Bondo site, women using DMPA received intramuscular injections at the study clinic during follow-up visits; injections were administered after blood was drawn. The DMPA used at the site was Depo-Progestin manufactured by PT Harsen in Indonesia, distributed by PSI and labeled as Femiplan in Kenya.

Plasma samples used for this analysis were drawn between July 2009 and March 2011. Samples were prepared and kept frozen at -20 °C for up to 33 months prior to shipment to PPD Development Labs (Richmond, VA, USA) where MPA levels were determined via a validated sensitive and selective high-performance liquid chromatography method coupled with tandem mass spectrometry (HPLC-MS/MS). The detection limit for MPA was 0.02 ng/mL and was determined during validation of the assay according to the vendor's standard operating procedure requirements. Back-calculated calibration standards, and intra-assay quality controls met acceptable precision and accuracy criteria (+/− 20%). In addition, prior to analysis of clinical samples, acceptable precision and accuracy runs were conducted in order to support continued use of the bioanalytical method. All correlation coefficients were > 0.997. Assay precision, expressed as the percent coefficient of variation (%CV), averaged 5.88%, 5.54%, 7.96%, 5.17% and 3.33% for the 0.05, 0.1, 0.3, 1.0, and 3.75 ng/mL quality control (QC) standards, respectively. Assay accuracy was expressed as the ratio (%) between the mean of the QC standard and the theoretical concentration of that standard resulting in inter-assay accuracy of 102.9–108.3%, with intra-assay accuracy between 98.8–115.5%.

To confirm adherence to study drug, levels of tenofovir (TFV) in plasma and tenofovir-diphosphate (TFV-DP) in upper-layer packed cells (ULPC) concentrations at four-week intervals had previously been measured [6]. For the current analysis, we defined “good” adherence as having both plasma TFV exceeding 10 ng/ml and ULPC TFV-DP exceeding 100,000 femtomoles, consistent with taking four or more doses of TDF/FTC per week [7].

We summarized MPA levels by study arm for up to two 12-week intervals. We evaluated the effects of TDF/FTC use (good adherence, non-zero adherence, or no adherence/placebo), baseline BMI (<=20 or > 20), age (18–24, 25–29, or 30 +) and previous MPA use (i.e., either presence of MPA in the plasma sample taken at the time of enrollment or second interval of use in sub-study) on MPA levels. For each participant contributing a complete set of data (i.e., injection visit and weeks 4, 8, and 12 post-injection), we used a linear trapezoidal procedure to compute a crude area under the curve (AUC) from the injection visit through the end of the 12-week dosing interval (we considered this a crude AUC measure due to sparse sampling at 4-week intervals, which also precluded making reasonable estimates of other PK parameters such as maximum concentration and time to maximum concentration). We estimated the crude AUC separately for women in interval 1 with no previous DMPA use, interval 1 with previous DMPA use, and interval 2. The effect of cumulative TDF/FTC use during the injection interval (number of 4-week periods with good adherence), MPA use in the previous interval, BMI, and age were then applied to log-transformed AUCs using a mixed effects linear model with random intercept term. We also calculated the proportion of women with values below the presumed contraceptive threshold for DMPA (0.1 ng/ml) [8], [9].

## 3 Results

We excluded interval 1 or interval 2 data from two women indicating either no injection had occurred (despite a data form entry to the contrary) or results strongly indicating that the injection was given before drawing of the 12-week blood sample, leaving 61 women contributing data to the first interval of DMPA use, 49 women contributing to a second interval of use, 62 women contributing to at least one interval, and a total of 110 intervals of use (65 Placebo, 45 TDF/FTC; Fig. 1).

In this sub-analysis, women in the placebo group were less likely to be married than women in the TDF/FTC group (72% versus 96%), but the two groups were otherwise similar in their demographic and physical characteristics at enrollment, including BMI (Table 1). Fifty two women (84%) reported using DMPA as their contraceptive method at screening, but only 42 (81%) had detectable MPA at enrollment. Prior dates of injections could not be consistently obtained, so we do not know how long the women with no evidence of MPA at enrollment had gone between injections.

**Fig. 2:**
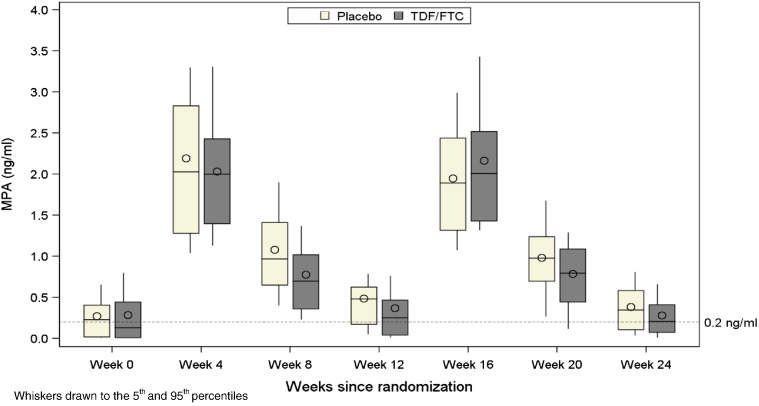
MPA levels over time since enrollment by treatment group. (N = 61 for weeks 0–12 and N = 49 for weeks 16–24).

Mean MPA levels in the initial interval of use were 2.13, 0.95, and 0.44 ng/mL at weeks 4, 8, and 12, respectively. The 12-week troughs were about 0.1 ng/mL lower in the TDF/FTC group, but the differences were not statistically significant (p = .07 for first interval, p = .34 for second interval, and p = .09 when accounting for repeated interval data) (Fig. 2).

Of 61 women with data following their first in-study DMPA injection, 16 (26.2%) had MPA levels < 0.1 ng/ml at their week 12 visit and 6 (9.8%) had undetectable MPA levels (Table 2). Similar results were apparent following their second in-study DMPA injection, with 13 of 49 women (26.5%) having MPA levels below 0.1 ng/ml twelve weeks after injection and 4 (8.2%) with undetectable levels.

BMI, previous use of DMPA, and use of TDF/FTC did not significantly affect log-transformed MPA values at weeks 4, 8, and 12 (results not shown). Although crude AUC values for MPA were higher in the placebo group than in the active group (Table 3), there was no significant effect of TDF/FTC use on log-transformed AUC values in a model that adjusted for actual TDF/FTC use (Table 4). In exploratory analysis, the geometric mean AUC ratio for injection intervals with at least one 4-week period with good-to-excellent TDF/FTC adherence versus intervals without any evidence of good TDF/FTC use (including women assigned placebo) was 0.91 (95% CI: 0.78–1.08) after adjusting for repeated measures on subjects. However, there were too few intervals with good-to-excellent adherence throughout the entire injection interval to explore this further.

**Fig. 3:**
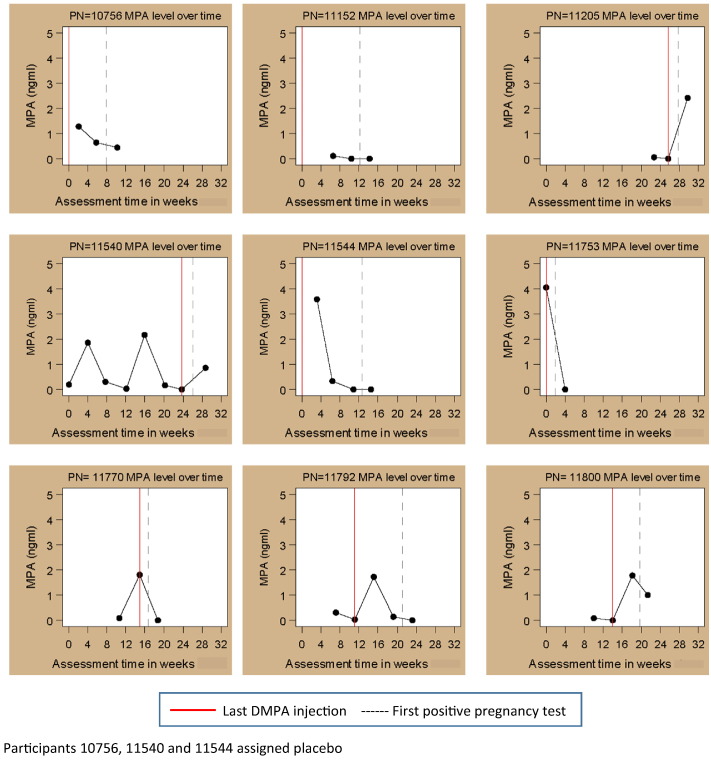
MPA levels among DMPA users who became pregnant during the FEM-PrEP trial.

Twelve women (seven assigned to TDF/FTC and five assigned to placebo) tested positive for pregnancy while on DMPA. Of the nine with specimens available for analysis, five had MPA levels < 0.15 ng/mL at the time of their last negative pregnancy test and four of those had levels below the detection limit (Fig. 3).

## 4 Discussion

In our sub-analysis of MPA levels among DMPA users in the Bondo, Kenya site of the FEM-PrEP study we found similar MPA levels across two DMPA dosing intervals among women assigned to TDF/FTC and placebo, adjusting for study drug adherence. While our results suggest that concomitant oral TDF/FTC use does not dramatically affect MPA levels among users of DMPA, we did not have high power to detect smaller than 50% reductions in trough concentrations. Also, we cannot exclude the possibility that any effect of TDF/FTC use was attenuated due to overall poor adherence. Even with these limitations, our finding of no evidence of a significant pharmacokinetic interaction is expected given that neither TDF nor FTC are cytochrome p450 enzyme inducers, and thus would be unlikely to increase MPA metabolism. Similar results were seen in a study of COC users where co-administration of TDF resulted in no change in ethinyl estradiol (EE) and norelgestromin levels [18]. Unexpectedly, we found noticeably lower MPA levels in both study groups at the end of the 12-week dosing interval than have been reported elsewhere [8], [10], [11], [12], [13]. More than a quarter of women had levels below the presumed contraceptive threshold of 0.1 ng/ml, and nearly 10% had levels below the assay detection limit.

The low MPA trough levels seen in our study are reflected in the relatively higher pregnancy rate among DMPA users in Bondo (3.1 per 100 woman-years) compared with DMPA users at the other FEM-PrEP sites (0.7 per 100 woman-years) that used another DMPA product, Petogen manufactured by Helm AG (Hamburg, Germany). The lowest MPA concentrations believed necessary to suppress ovulation ranges from 0.1 to 0.2 ng/mL [8], [9] While this threshold level is based on very limited data and prior studies have shown that MPA concentrations can vary quite dramatically between individuals, most published data indicate levels at three months remain well above 0.2 ng/mL [8], [10], [11], [12], [13]. Furthermore, while the early studies on which the threshold are based measured MPA using radioimmunoassay (RIA) which is known to produce higher results due to cross-reactivity with metabolites, we found lower levels than studies that measured MPA using a comparable mass spectrometry assay [15], [16].

The fact that numerous women in this sub-study had levels below 0.1 ng/ml at the end of their 12-week injection intervals raises a number of questions, including whether it is appropriate to assume that MPA pharmacokinetics and pharmacodynamics are sufficiently uniform across populations. Few studies compare MPA levels across populations and published data are conflicting. While older PK studies of intramuscular DMPA among women in Mexico and Thailand found more rapid disappearance of MPA and return to ovulation among Thai women compared with Mexican women, newer studies of subcutaneous DMPA show no racial differences [16], [19]. Furthermore, the only available data from Africa show no difference in trough MPA levels between Black African, Indian and White South African women [10].

It has been postulated that inter-individual differences in MPA metabolism may be attributable to weight or body mass index (BMI) [20], [21], [22], but we found no significant effect of BMI on MPA levels. Also, only one woman who became pregnant in our analysis would be considered even slightly overweight.

Inter-individual differences in pharmacogenetics can also have implications for drug pharmacokinetics, efficacy, and dosage. Recent studies designed to map pharmacogenetic traits among African populations show considerable variation in allele frequencies known to be associated with drug metabolism [23], [24]. A comprehensive review of injectable contraception published in 1981 suggested that population differences in the metabolism of contraceptive steroids including DMPA is likely [25], [26]; over thirty years later the question of whether and how DMPA metabolism varies across populations remains unanswered.

While we cannot rule out the possibility that the low levels of MPA measured in this study were the result of problems with the handling and/or provision of the DMPA at the study site, study staff and study records indicated that the DMPA vials were stored properly, re-suspended per instructions, and that drug caking was not observed. Injections were given by trained nurses with prior experience providing DMPA. Plasma samples were kept frozen at the recommended temperature for up to 33 months. While this is longer than the 722 day stability data reported by PPD, contraceptive steroids such as MPA are very stable and likely not affected by the length of storage in this study. It is always possible that mishandling of the samples could have occurred either at the study site, in the shipping process or at the assaying lab. However, no evidence of such problems was documented.

Another possible explanation for our unusual results is that the DMPA product used at the Bondo site (Femiplan) is not equivalent to other marketed DMPA products. Given that FEM-PrEP ended nearly four years ago, we were unable to test samples from the DMPA product lots used during the study. However, we did do several quality assurance tests on two unexpired samples (manufactured in March 2011 and May 2013) of the same product (Femiplan, distributed by PSI, manufactured by PT Harsen). Measurement of percent label claim of MPA per vial and pH (data not shown) revealed the Femiplan samples to be in compliance with specifications. However, we noted that the proportion of MPA particles greater than 10 µm was greater than what we had observed with other DMPA products. Furthermore, the 2011 Depo-Progestin sample had a greater proportion of larger particles than the 2013 sample. It has been theorized that the particle size of DMPA in various formulations may play a role in duration of action and contraceptive efficacy [19], [27]. However, in prior studies, *smaller* particle size was associated with more rapid absorption. A larger DMPA particle size might be expected to have had an opposite effect on PK profiles. It is possible that the larger particle size may have led to agglomeration or clumping, which could potentially affect syringeability and prevent injection of the full dose.

Strengths of our study include a simple design and use of a validated, highly sensitive HPLC-MS/MS MPA assay, the same as that used for MPA analysis in studies conducted by Pfizer which supported US FDA approval of Depo-SubQ Provera 104 [14], as well as for several other PK studies [15], [16], [17]. Additionally, our findings add to the exceedingly limited data on DMPA pharmacokinetics in Africa. However, we could only evaluate blood levels at 4 week intervals, limiting our ability to robustly assess PK of MPA. Given the widespread use of DMPA and the large number of DMPA products available worldwide, our findings of lower than expected MPA levels among DMPA users with documented injections highlights the need for more data confirming effectiveness of different products in different populations. Such data should include post-marketing surveillance data on pregnancy rates among users of the various products, PK data of various DMPA products in different settings, and chemical/physical tests for product quality assurance.

## Figures and Tables

**Table 1:** Demographic characteristics at baseline among women included in the sub-study.

Characteristic	Placebo N = 36	TDF/FTC N = 26	Total N = 62
Age (in years)			
18–24	36.1%	7.7%	24.2%
25–29	22.2%	38.5%	29.0%
30–35	41.7%	53.8%	46.8%
Median (Range)	28 (19–35)	30 (22–34)	29 (19–35)
Height (cm)			
Mean (SD)	167.6 (6.9)	165.0 (6.0)	166.5 (6.6)
Median (Range)	167 (146–180)	165 (152–177)	166 (146–180)
Weight (kg)			
Mean (SD)	60.6 (9.2)	58.5 (6.7)	59.7 (8.3)
Median (Range)	59 (39–81)	59 (41–71)	59 (39–81)
BMI			
Mean (SD)	21.6 (3.0)	21.5 (2.6)	21.5 (2.8)
Median (Range)	21 (17–29)	22 (16–29)	21 (16–29)
Years of school completed			
<= 9	80.6%	88.5%	83.9%
> 9	19.4%	11.5%	16.1%
Median (Range)	8 (0–12)	8 (0–12)	8 (0–12)
Marital Status			
Not married, not living with man	27.8%	3.8%	17.7%
Married, not living with man	2.8%	3.8%	3.2%
Married, living with man	69.4%	92.3%	79.0%
Ever been pregnant			
Yes	100%	100%	10%
Number of pregnancies			
Median (Range)	3 (1–7)	4 (1–6)	3 (1–7)
Contraceptive used at screening			
Oral contraceptives	8.3%	3.8%	6.5%
Injectable	80.6%	88.5%	83.9%
None	11.1%	7.7%	9.7%

**Table 2:** MPA levels at the end of the injection interval (week 12) by study arm.

	N	Mean (95% CL)	% < 0.2 ng/ml	% < 0.1 ng/ml	% < 0.02 ng/ml
Interval 1					
Placebo	35	0.49 (0.40, 1.27)	28.6	20.0	5.7
TDF/FTC	26	0.37 (0.25, 1.99)	46.2	34.6	15.4
Pooled	61	0.44 (0.39, 1.14)	36.1	26.2	9.8
Interval 2					
Placebo	30	0.39 (0.31, 1.17)	30.0	23.3	6.7
TDF/FTC	19	0.28 (0.19, 1.22)	47.4	31.6	10.5
Pooled	49	0.35 (0.30, 0.84)	36.7	26.5	8.2

**Table 3:** AUC_0–12 week_ by injection interval and treatment group.

	N	Mean (95% CL)
First interval, no previous use		
Placebo	8	81.9 (67.4, 106.3)
TDF/FTC	9	76.2 (65.3, 92.5)
First interval, previous use		
Placebo	27	106.4 (91.9, 127.2)
TDF/FTC	16	95.8 (77.7, 125.9)
Second interval		
Placebo	29	93.7 (84.8, 105.5)
TDF/FTC	19	91.8 (77.6, 113.9)

**Table 4:** Effect of Cumulative TDF/FTC use in 4-week periods on log-transformed AUC values.

	Estimate (95% CI)	P-value^2^
Cumulative TDF/FTC Use^1^	− 0.00 (− 0.08, 0.07)	0.904
Previous MPA use: Yes Vs No	0.11 (− 0.07, 0.29)	0.219
BMI > 20 Vs < = 20	0.07 (− 0.09, 0.24)	0.365
Age in years (18–24 as reference)		
30 +	− 0.06 (− 0.25, 0.13)	0.530
25–29	− 0.24 (− 0.46, − 0.02)	0.030

1 Cumulative TDF/FTC use in the injection window (0–3).

2 P-value based on mixed linear model with random effect as intercept.
